# Distinct strategies of epithelial cell barrier disruption by *Leptospira interrogans* isolated from human patients in Okinawa, Japan

**DOI:** 10.1371/journal.pntd.0013693

**Published:** 2025-11-04

**Authors:** Tetsuya Kakita, Claudia Toma, Isabel Sebastián, Hunter Barbee, Tetsu Yamashiro

**Affiliations:** 1 Graduate School of Medicine, Department of Bacteriology, University of the Ryukyus, Okinawa, Japan,; 2 Research Center for Infectious Disease, Okinawa Prefectural Institute of Health and Environment; Cornell University, UNITED STATES OF AMERICA

## Abstract

**Background:**

Leptospirosis is a bacterial infection common in tropical and subtropical regions, which causes feverish conditions. Although approximately half of human leptospirosis cases in Japan are reported in Okinawa, a subtropical area, the pathogenic mechanisms of clinical isolates from this region remain unknown. This study aimed to identify the infection mechanisms of *L. interrogans* isolates from Okinawa (Oki-strains) in human renal proximal tubule epithelial cells (RPTECs).

**Methodology/principal findings:**

The transepithelial electrical resistance (TEER) measurements of 11 Oki-strains in infected renal proximal tubule epithelial cells (RPTECs) showed that all strains caused a decrease in TEER by 48 hours post-infection. Imaging analysis of RPTECs infected with two selected strains (Oki53 and Oki65) revealed that both strains induced the displacement of adherens junction (AJ) proteins E-cadherin, α-catenin, afadin, and nectin-2 and cytoskeletal F-actin disorganization. However, western blotting analysis revealed that AJ protein levels were not reduced, except for afadin—an important protein for linking F-actin to AJs. Chemical inhibition revealed that the proteosome inhibitors MG132 and bortezomib and pan-caspase inhibitor Z-VAD-FMK prevented the Oki53-induced TEER decrease, AJ protein mislocalization, afadin degradation, and F-actin disorganization. However, in Oki65-infected RPTECs, the inhibitors partially prevented these effects. Thus, the AJ-F-actin link and epithelial barrier were fully preserved in Oki53-infected RPTECs pretreated with these inhibitors at 24 h post-infection (~TEER 130% of initial TEER), whereas in Oki65-infected cells, the AJ-F-actin link was maintained only partially (~TEER 70%).

**Conclusions/significance:**

Our findings suggest that the maintenance of epithelial barrier integrity requires both the afadin-F-actin and α-catenin-F-actin links, and that Oki53 and Oki65 employ distinct strategies to disrupt the AJ-F-actin link and, thus, the epithelial barrier.

## Author summary

Leptospirosis is a bacterial infection common in tropical and subtropical areas, including Okinawa, Japan. Pathogenic *Leptospira* colonize animal kidneys and are released in urine. In humans, colonization sometimes causes kidney failure. This study aimed to identify the infection mechanisms in *L. interrogans* isolates from Okinawa on human renal proximal tubule epithelial cells (RPTECs). All 11 clinical isolates induced a reduction in transepithelial electrical resistance (TEER), an indicator of cell-cell adhesion strength, in infected RPTECs by 48 h post-infection. Moreover, two selected strains, Oki53 and Oki65, induced adherens-junction protein displacement (afadin, α-catenin, E-cadherin, and nectin-2), afadin degradation, and cytoskeleton F-actin disorganization. In addition, proteasomal and pan-caspase inhibitors completely prevented these effects in Oki53-infected RPTECs, but only partially prevented the TEER decrease and afadin degradation in Oki65-infected cells. Consequently, the connection between junction proteins and the cytoskeleton (the AJ–F-actin link) and the overall epithelial barrier were fully preserved in Oki53-infected cells when pretreated with these inhibitors, but only partially preserved in Oki65-infected cells. These findings suggest that maintaining the epithelial barrier integrity depends on both the afadin–F-actin and α-catenin–F-actin links, and that different strains of *Leptospira* disrupt this barrier via different strategies.

## 1. Introduction

Human leptospirosis, a globally prevalent zoonosis, caused by pathogenic *Leptospira* spp., with approximately 1 million cases and 58,900 deaths reported annually [1]. The genus *Leptospira* comprises 69 species, 25 serogroups, and over 300 serovars, which are classified into either pathogenic or non-pathogenic clades [2,3]. Among these, highly virulent *Leptospira* species (also called the P1 + clade) such as *L. interrogans*, *L. borgpetersenii*, and *L. kirschneri* are well-known causative agents of human leptospirosis worldwide [4,5]. Pathogenic *Leptospira* spp. colonize the proximal renal tubules of maintenance hosts such as wild animals, livestock, and companion animals and are excreted in their urine [6]. As incidental hosts, humans become infected upon direct contact with the urine or exposure to water or soil contaminated with it [6,7]. In Japan, 16–76 cases of human leptospirosis were reported annually between 2004 and 2024, with about half of the cases occurring in Okinawa Prefecture, a subtropical region in Japan [8]. The leptospirosis cases in Okinawa are predominantly associated with exposure to rivers during recreational or occupational activities in the summer, particularly in the northern part of the Okinawa main island and the Yaeyama region [8]. Among pathogenic *Leptospira* strains isolated from human patients in Okinawa, *L. interrogans* serogroup Hebdomadis accounts for approximately half, followed by *L. interrogans* serogroups Autumnalis and Pyrogenes [8]. Although the serogroup Hebdomadis is not a major causative agent in other countries. In Japan, *L. interrogans* serogroup Hebdomadis is associated with mild and severe clinical symptoms, including renal and hepatic dysfunction, comparable to other serogroups, necessitating a cautious treatment approach [8]. The clinical manifestations of leptospirosis are diverse, and clarifying its infection mechanisms may contribute to improved treatment and prevention of severe cases.

The epithelial cell barrier is maintained apically by tight junctions (TJs), followed by the more basal adherens junctions (AJs), and the most basal desmosomes. At AJs, the extracellular domain of E-cadherin is believed to be crucial in intercellular adhesion, with its intracellular domain interacting with β-catenin and α-catenin, ultimately linking to F-actin, a major cytoskeletal component [9]. In addition, the transmembrane protein nectin is also an important cell–cell adhesion molecule at AJs that links the AJs to the actin cytoskeleton through afadin [10]. In 2024, Mangeol et al. reported that the E-cadherin–catenin and Nectin–Afadin complex form distinct layers at AJs and observed that F-actin predominantly localizes to the Nectin–Afadin complex layer in mature epithelia, highlighting the critical role of the Nectin–Afadin complex in the intercellular barrier [9].

*Leptospira* spp. infect the body either percutaneously or permucosally, disseminates hematogenously, and proliferates in the renal proximal tubules, where it forms biofilms for colonization [11]. Previous studies have shown that infection with the pathogenic strain *Leptospira interrogans* serogroup Pyrogenes serovar Manilae strain UP-MMC-NIID (LM) reduces the transepithelial electrical resistance (TEER) of renal proximal tubule epithelial cells (RPTECs) [12,13]. This strain also induces endocytosis of E-cadherin at AJs, leading to its depletion from the plasma membrane and subsequent disorganization of cytoskeletal F-actin [12,13]. These results indicate that pathogenic *Leptospira* disrupts the epithelial barrier; however, the infection mechanism of *L. interrogans* clinical isolates in Okinawa, Japan, remains unknown [8]. Therefore, this study aimed to identify the mechanisms by which *Leptospira* strains isolated from human leptospirosis cases in Okinawa disrupt the epithelial cell barrier of RPTECs.

## 2. Methods

### 2.1 Bacterial cultures and strains

In accordance with the Infectious Diseases Control Law of Japan, the Okinawa Prefectural Institute of Health and Environment routinely receives specimens for the laboratory confirmation of clinically suspected leptospirosis. Notably, 11 pathogenic *Leptospira interrogans* strains (Oki-strains) were isolated in Ellinghausen-McCullough-Johnson-Harris (EMJH) broth from blood samples, between June and October 2023 (Table 1). When the bacterial culture from patients reached the late exponential growth phase, several vials for each strain were stocked at -80°C in culture medium supplemented with 2.5% DMSO to maintain their virulence. *L. interrogans* serovar Manilae strain UP-MMC-NIID (LM) [14] was used as the positive control of cell barrier disruption and *L. biflexa* serovar Patoc strain Patoc I as the non-pathogenic negative control. During this study, pathogenic strains, including LM, were cultured at 30°C, stationary in EMJH broth with no more than five passages to maintain reproducible virulence. For cell infection experiments, a stationary growth-bacterial culture was diluted in fresh EMJH and cultured for 3 days with agitation at 30°C.

**Table 1:** *Leptospira interrogans* strains isolated from patients in Okinawa, Japan, in 2023.

Strain No.	Abbreviations	Serogroup	Estimated area of infection
L238039	Oki39	Hebdomadis	Iriomote
L238047	Oki47	Hebdomadis	Ishigaki
L238049	Oki49	Hebdomadis	Iriomote
L238051	Oki51	Hebdomadis	Ishigaki
L238053	Oki53	Pyrogenes	Iriomote
L238057	Oki57	Hebdomadis	Iriomote
L238065	Oki65	Hebdomadis	Iriomote
L238067	Oki67	Pyrogenes	Iriomote
L238085	Oki85	Hebdomadis	Unknown
L238086	Oki86	Pyrogenes	Yonaguni
L238092	Oki92	Hebdomadis	Iriomote

### 2.2 Cell culture

RPTEC/TERT1 (American Type Culture Collection, ATCC CRL-4031) cells, human RPTECs immortalized by human Telomerase Reverse Transcriptase, were grown in Dulbecco’s Modified Eagle Medium/Nutrient Mixture F-12 (DMEM/F-12; Gibco, ThermoFisher, Waltham, MA, USA) supplemented with 5 pM triiodothyronine, 10 ng/mL recombinant human epidermal growth factor, 3.5 μg/mL ascorbic acid, 5 μg/mL transferrin, 5 μg/mL insulin, 8.65 ng/mL sodium selenite and 100 μg/mL G418. Further, the cells were seeded at a density of 1 × 10^6^ cells/well in polyethylene terephthalate hanging cell culture inserts with a pore size of 3 μm (Falcon; Corning, New York, NY, USA) in the upper chamber of a Falcon Companion six-well tissue culture plate (Corning, NY, USA). Cells were maintained in a humidified incubator at 37°C with 5% CO_2_ for 14 days. The medium was exchanged every 2 days to facilitate monolayer maturation. The TEER of the monolayers was measured using a Millicell-ERS cell resistance indicator (MilliporeSigma, Burlington, MA, USA). After subtracting the value of a cell-free insert (blank), the mean TEER value was expressed as Ωcm^2^. The TEERs of cells before infection were designated as the baseline values. The percentage TEER, relative to the baseline value, was calculated using the following formula: (TEER of experimental wells/baseline TEER of experimental wells) ×100%.

### 2.3 Cell infection

After being cultured on inserts for 14 days, the RPTECs had their medium replaced with supplement-free DMEM/F-12 and were then infected with *Leptospira* at a multiplicity of infection of 100 from the basolateral side. In experiments with inhibitors, they were added 30 min before infection. The inhibitors used were 10 μM MG-132 (a proteasome inhibitor; Sigma-Aldrich, St. Louis, MI, USA), 200 nM bortezomib (a proteasome inhibitor; FUJIFILM Wako, Osaka, Japan), 20 μM benzyloxycarbonyl-Val-Ala-Asp (OMe)-fluoromethylketone (Z-VAD-FMK, a pan-caspase inhibitor; R&D Systems, Minneapolis, MN, USA). As a control, cells were treated with DMSO, the solvent for these inhibitors. Cells were subsequently incubated at 37°C with 5% CO_2_ during infection and fixed for immunostaining or lysed for immunoblotting.

### 2.4 Immunoblotting

RPTECs were lysed using radioimmunoprecipitation assay buffer (Nacalai, Kyoto, Japan; 50 mM Tris-HCl buffer [pH 7.6], 150 mM NaCl, 1% Nonidet P40, 0.5% sodium deoxycholate, and 0.1% sodium dodecyl sulphate), supplemented with a protease inhibitor cocktail (Nacalai Tesque, Kyoto, Japan). The cell lysates were collected with a cell scraper, mixed with Laemmli sample buffer [15], heated for 10 min at 100 °C, and sonicated for 3 min (5 s sonication with 5 s intervals). The samples were subjected to protein separation using precast mini-Protean TGX 4–15% gel (Bio-Rad Laboratories, Hercules, CA, USA) and further processed for immunoblots.

Primary antibodies used for immunoblotting included mouse monoclonal antibodies: anti-α-catenin (1:1,000; sc-9988), anti-E-cad (1:1,000; # 610182, BD Transduction Laboratories, Franklin Lakes, NJ, USA, BD), anti-afadin (1:500; #610732, BD), and anti-glyceraldehye 3-phosphate dehydrogenase (GAPDH) (1:2,500; sc-32233); and rabbit monoclonal anti-nectin-2 (1:2,000; CST#95333, Cell Signaling Technology, Danvers, MA, USA). The secondary antibodies were a horseradish peroxidase (HRP)-anti-rabbit IgG antibody (1:25,000; #111-035-144, Jackson ImmunoResearch (JIR), West Grove, PA, USA) and HRP-anti-mouse IgG antibody (1:7,500; JIR#715-005-150). Afterward, the Amersham ImageQuant 800 Imaging System (GE Healthcare, Chicago, IL, USA) was used to visualize the protein bands. Band intensities in immunoblots were analyzed using ImageJ software (version 1.53). Reacting protein bands were normalized with GAPDH as a loading control, and the relative expression level of each protein was calculated by considering the ratio of the analyzing protein/GAPDH in non-infected RPTECs as one.

### 2.5 Immunostainings

RPTECs were fixed in cold methanol for 15 min, permeabilized, and blocked with buffer A (5% bovine serum albumin [BSA], 1% Triton X-100 in Tris-buffered saline [TBS]; 50 mM Tris and 150 mM NaCl [pH 7.4]) for 15 min to analyze the localization of AJ complex proteins. For F-actin staining, infected cells were fixed with 2% paraformaldehyde in phosphate-buffered saline for 2 h at 4°C to retain the quaternary protein structure, which is necessary for phalloidin binding, and washed twice with TBS. The filter membranes were detached from the hanging culture inserts before immunostaining.

In addition, antibodies were diluted in TBS containing 1% BSA and 0.1% Triton X-100. The primary antibodies for RPTEC proteins were rabbit monoclonal antibodies: anti-E-cad (1:50; CST#3195) and anti-nectin-2 (1:50; CST#95333); and mouse monoclonal antibodies: anti-α-catenin (1:50; sc-9988) and anti-afadin (1:100; #610732, BD). F-actin was stained with a 1:200 dilution of rhodamine phalloidin (Abcam, Cambridge, UK). Cells were counterstained to label the DNA with a 1:50 dilution of TO-PRO-3 (Invitrogen, Waltham, MA, USA). Secondary antibodies used for immunofluorescence analysis included anti-rabbit IgG Alexa 488- (1:100; JIR#711-545-152) and anti-mouse IgG Alexa 488-conjugated antibody (1:100; JIR#715-545-151) or Cy3-conjugated antibody (1:100; JIR#715-165-151). After mounting with SlowFade Diamond Antifade Mountant (Invitrogen), the compiled Z-stack images were acquired using a Leica TCS-SPE confocal laser scanning microscope with LEICA LAS AF acquisition software (version 2.6.0.7266, Leica Microsystems CMS GmbH, Germany).

The fluorescence intensity of proteins at cell–cell junctions was quantified from maximum projections of Z-stack images using ImageJ software (version 1.53). The “Find Edges” function was applied to enhance junctional signals, followed by thresholding to distinguish the fluorescence signal from the background. The integrated signal intensity was normalized by the number of cells within each field. The relative fluorescence intensity was calculated using the values of non-infected RPTECs pretreated with DMSO as 1.

### 2.6 Statistical analyses

Statistical significance was determined using an unpaired two-tailed Student *t*-test. All the data were presented as the mean of at least three independent determinations per experimental condition. Differences were considered significant at a *p-*value of < 0.05.

## 3. Results

### 3.1 *Leptospira interrogans* from Okinawa (Oki-strains) induce epithelial TEER reduction

**Fig 1:**
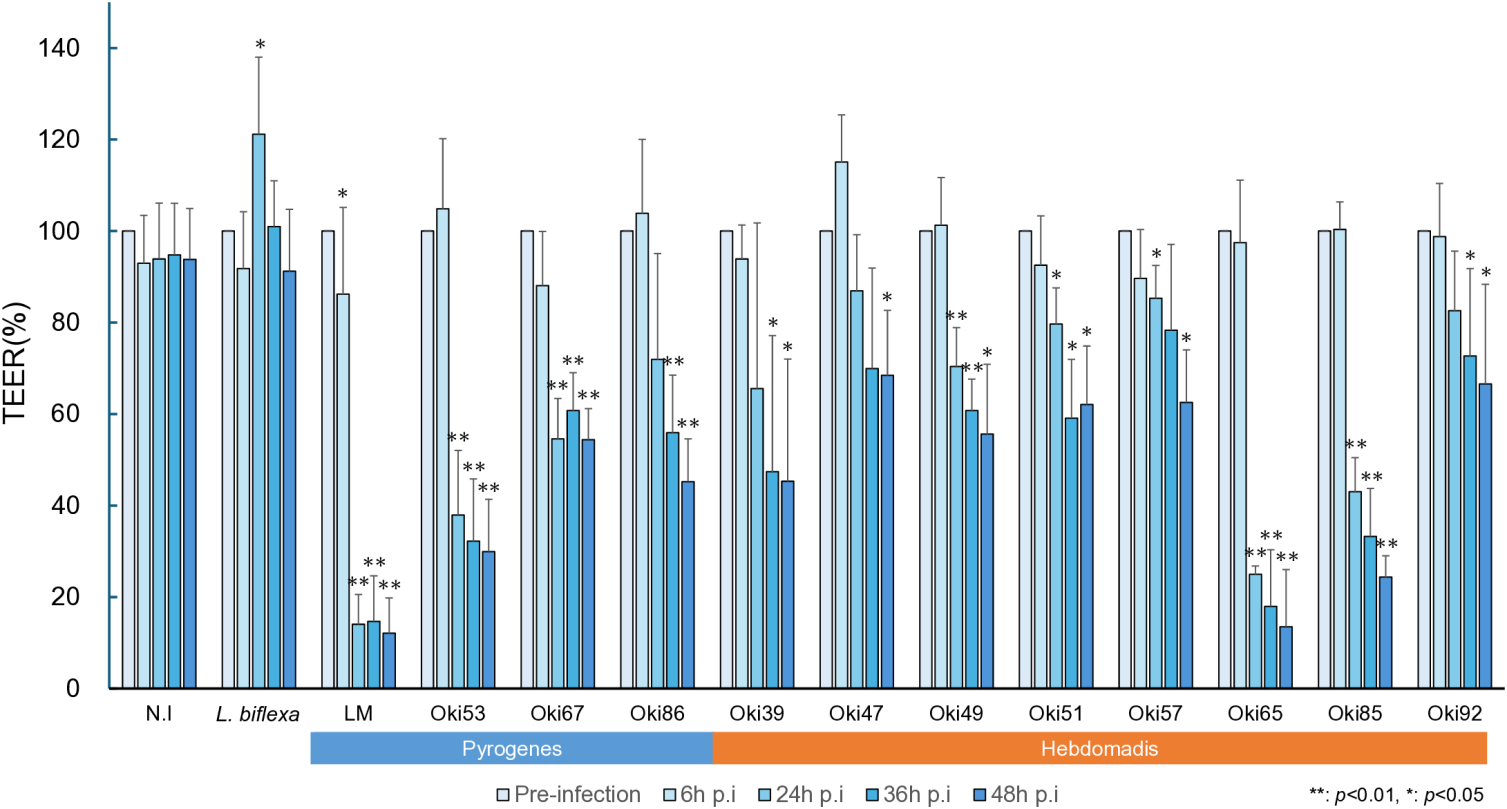
*Leptospira interrogans* from Okinawa induces epithelial barrier disruption with different kinetics. Epithelial barrier integrity was assessed by measuring the TEER of RPTECs at pre-infection and 6, 24, 36, and 48 h post-infection. TEER values were expressed as percentages relative to the pre-infection value for each strain and are presented as the mean ± standard deviation from at least three independent transwells. Statistical significance was determined by comparing each time point to the corresponding pre-infection value. *p < 0.05 and **p  < 0.01. Blue and orange bars below the isolates represent the serogroups of the respective strains.LM, *L. interrogans* serovar Manilae strain UP-MMC-NIID; N.I., not infected, RPTECs, renal proximal tubule epithelial cells; SD, standard deviation; TEER, transepithelial electrical resistance.

The highly pathogenic *L. interrogans* serovar Manilae strain UP-MMC-NIID (LM) disrupts the epithelial cell barrier within 24 h [12,13]. To determine whether the disruption of epithelial cell barrier is an intrinsic characteristic among clinical isolates, we infected RPTECs with 11 Okinawan *L. interrogans* isolates, LM, and a non-pathogenic *L. biflexa* strain. The TEER measurements of non-infected RPTECs during 48 h did not show significant fluctuations (Fig 1). TEER measurement of infected cells was performed at 6, 24, 36, and 48 h post-infection (p.i). Seven strains (Oki53, 67: serogroup Pyrogenes; Oki49, 51, 57, 65, 85: serogroup Hebdomadis) showed a significant decrease of TEER (**p* *< 0.05) at 24h p.i., whereas the remaining four strains (Oki86: serogroup Pyrogenes; Oki39, 47, 92: serogroup Hebdomadis) showed different kinetics of TEER decrease. All 11 strains caused a significant decrease in TEER within 48 h p.i, varying from 13.5–68.4%. Notably, TEER was significantly increased (**p* *< 0.05) in *L. biflexa*-infected RPTECs at 24 h p.i. These findings suggest that clinical isolates from Okinawa disrupt the epithelial cell barrier, regardless of the serogroup. Different kinetics between strains may indicate that various mechanisms are involved.

### 3.2 Protein degradation inhibitors differentially prevent the disruption of the epithelial cell barrier by the clinical *Leptospira* isolates

**Fig 2:**
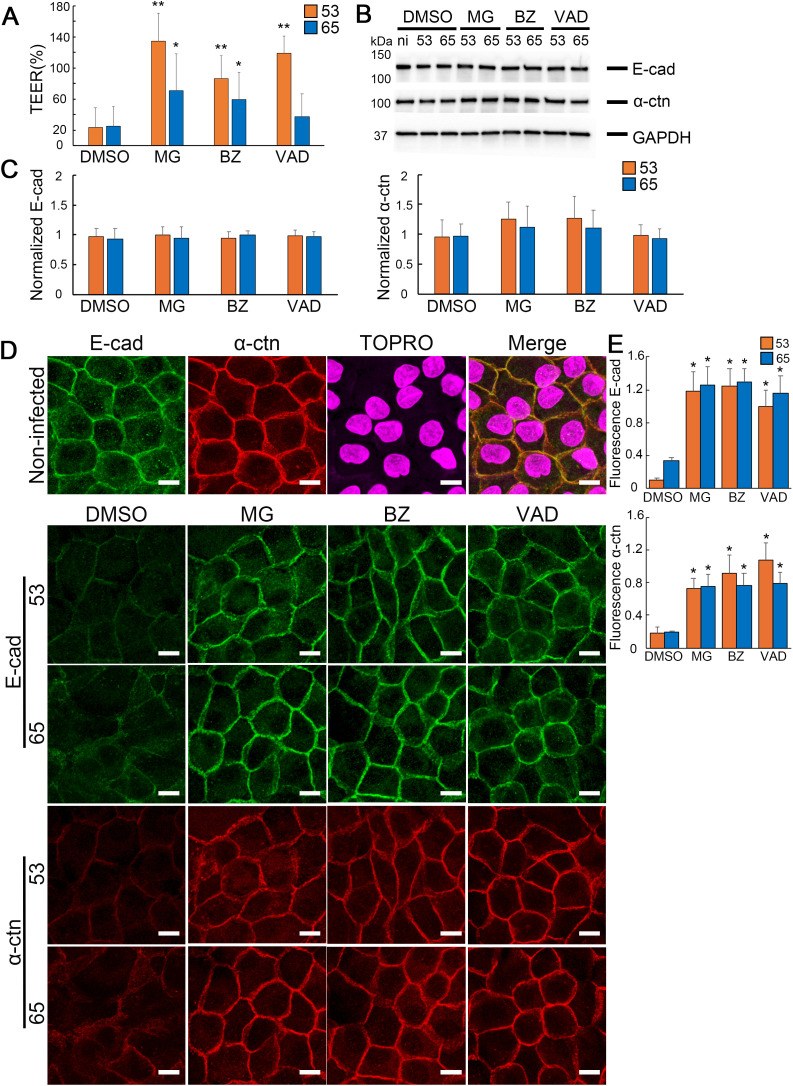
Proteosomal and pan-caspase inhibitors differentially prevent the disruption of the epithelial cell barrier by the clinical *Leptospira* isolates. RPTECs were pre-treated for 30 min with DMSO, MG132 (MG), bortezomib (BZ), and Z-VAD-FMK (VAD). (A) TEER measurements at 24 h p.i. Orange bars indicate TEER in RPTECs infected with Oki53, and blue bars indicate TEER in RPTECs infected with Oki65. TEER values in RPTECs pretreated with MG, BZ, and VAD were compared to the corresponding DMSO-pretreated RPTECs infected with each isolate. *p < 0.0*5* and **p < 0.01. (B) Whole-cell lysates were subjected to western blotting for E-cadherin (E-cad) and α-catenin (α-ctn) at 24 h p.i. The blot shown in panel (B) was cropped from the full blot shown in S1 Fig. (C) Normalized E-cad and α-ctn levels calculated by considering the ratio of analyzing protein/GAPDH in non-infected RPTECs as one. Each bar represents the mean ± standard deviation of at least three independent experiments. (D) Representative immunofluorescence images of E-cad (green) and α-ctn (red) staining in RPTECs. RPTECs were pre-treated for 30 min with DMSO, MG, BZ, or VAD, infected with clinical isolates, fixed with methanol at 24 h p.i., and processed for immunostaining. E-cad was stained with an Alexa Fluor 488-labeled antibody, and α-ctn with a Cy3-labeled antibody. The cell nuclei were stained with TO-PRO-3 (magenta). Scale bar: 10 μm. Merged images of E-cad, α-ctn, and TO-PRO-3 nuclear staining are shown in S2 Fig. (E) Relative fluorescence intensity of E-cad and α-catenin, considering the intensity of non-infected RPTECs pretreated with DMSO as 1. Each bar represents the mean ± standard deviation of three independent images. The relative fluorescence intensity of RPTECs pretreated with MG, BZ, and VAD was compared with that of RPTECs pretreated with DMSO for each strain. *p < 0.05 and **p < 0.01. ni*,* non-infected; TEER, transepithelial electrical resistance; RPTECs, renal proximal tubule epithelial cells; p.i, post-infection; DMSO, dimethyl sulfoxide.

We selected two *L. interrogans* strains, Oki53 (serogroup Pyrogenes) and Oki65 (serogroup Hebdomadis), which caused significant TEER reduction at 24 h p.i., to analyze their epithelial barrier disruption mechanisms. To characterize strain-specific differences, we used the proteasome inhibitors MG132 and bortezomib, and the pan-caspase inhibitor Z-VAD-FMK, which reportedly inhibit epithelial barrier disruption by *L. interrogans* serovar Manilae [13]. Pretreatment with the inhibitors markedly suppressed the Oki53-induced TEER decrease, maintaining 85.9–134.0% of baseline TEER (Fig 2A). In contrast, Oki65-induced TEER reduction was only partly prevented, with TEER maintained at 70.9% (MG132), 59.1% (bortezomib), and 37.6% (Z-VAD-FMK) (Fig 2A). These results suggest that the pathways responsible for TEER decrease, and hence epithelial barrier disruption, differ between Oki53 and Oki65. Notably, Oki53 appears to disrupt the epithelial barrier by hijacking host proteasome- and caspase-dependent pathways.

Studies have shown that LM induces E-cadherin displacement from the plasma membrane without decreasing total protein levels of E-cadherin or α-catenin [12]. As a potential difference in the mechanisms of epithelial barrier disruption between Oki53 and Oki65, we hypothesized that Oki53 does not decrease E-cadherin protein levels, whereas Oki65 may induce the decrease of E-cadherin and/or its linker protein α-catenin, which connects E-cadherin to F-actin. However, immunoblotting analysis of Oki53- or Oki65-infected RPTECs at 24 h p.i. revealed that neither strain affected total E-cadherin or α-catenin protein levels (Fig 2B and 2C). As AJ protein levels may remain unchanged despite alterations in their localization during infection, we analyzed their localization by immunofluorescence. In cells lacking E-cadherin, α-catenin homodimers can localize to the plasma membrane and interact with the actin cytoskeleton to promote cell adhesion [16]. Therefore, we performed immunofluorescence analysis of Oki53- and Oki-65-infected RPTECs at 24 h p.i. to assess subcellular E-cadherin and α-catenin localization. Both Oki53 and Oki65 induced E-cadherin and α-catenin displacement from the cell membrane (Fig 2D and 2E). Furthermore, RPTEC pre-treatment with MG132, bortezomib, or Z-VAD-FMK significantly prevented E-cadherin and α-catenin displacement in both strains (Fig 2D and 2E). These findings suggest that both Oki53 and Oki65 induce E-cadherin and α-catenin displacement from the epithelial cell membrane via proteasome- and caspase-dependent pathways. However, because TEER reduction by Oki65 was only partially prevented by these inhibitors, additional, distinct mechanisms beyond disruption of the E-cadherin–catenin complex possibly contribute to epithelial barrier disruption in this strain.

### 3.3 *L. interrogans* induce afadin degradation in infected RPTECs through different strain-dependent mechanisms

**Fig 3:**
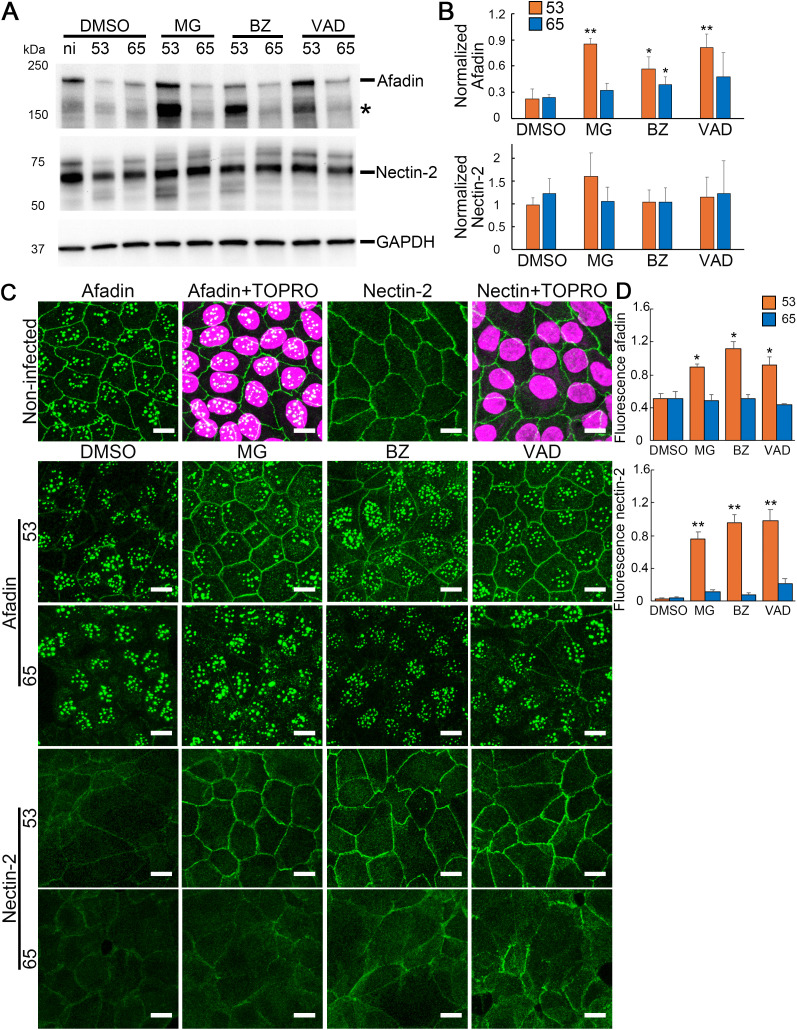
*L.* *interrogans* induces afadin degradation in infected RPTECs through different strain-dependent mechanisms. RPTECs were pre-treated for 30 min with MG132 (MG), bortezomib (BZ), and Z-VAD-FMK (VAD). (A) Whole-cell lysates were subjected to western blotting for afadin and nectin-2 at 24 h p.i. A fragment that was increased by adding inhibitors is denoted by an asterisk. *ni* indicates non-infected. The blots shown in panel (A) was cropped from the full blots shown in S3 Fig. (B) Normalized afadin and nectin-2 levels calculated by considering the ratio of analyzing protein/GAPDH in non-infected RPTECs as one. Each bar represents the mean ± standard deviation of at least three independent experiments. The effect of MG, BZ, and VAD was evaluated by comparing protein levels in RPTECs infected with each isolate, with or without inhibitor treatment. *p < 0.05 and **p < 0.01. (C) Representative immunofluorescence images of afadin and nectin-2 (green) staining in RPTECs. RPTECs were pre-treated for 30 min with DMSO, MG, BZ, or VAD, infected with clinical isolates, fixed with methanol at 24 h p.i., and processed for immunostaining. Afadin and nectin-2 were stained with an Alexa Fluor 488-labeled antibody. The cell nuclei were stained with TO-PRO-3 (magenta). Scale bar: 10 μm. Merged images of afadin with TO-PRO-3, and nectin-2 with TO-PRO-3, are shown in S4 Fig. (D) Relative fluorescence intensity of afadin and nectin-2, considering the intensity of non-infected RPTECs pretreated with DMSO as 1. Each bar represents the mean ± standard deviation of three independent images. The relative fluorescence intensity of RPTEC pretreated with MG, BZ, and VAD was compared with that of RPTEC pretreated with DMSO for each strain. *p < 0.05 and **p < 0.01. RPTECs, renal proximal tubule epithelial cells; DMSO, dimethyl sulfoxide; p.i., post-infection.

The connection between AJ proteins and F-actin is essential for maintaining a strong epithelial cell barrier; however, the inherent weakness of the E-cadherin–catenin complex and F-actin bond necessitates enhancement by binding partners such as afadin [17]. Based on our findings, we hypothesized that Oki65 disrupts the nectin–afadin complex, thereby affecting AJs and the supporting cytoskeleton, whereas Oki53 does not. To test this, immunoblotting and immunofluorescence analyses of afadin and nectin-2 in Oki53- or Oki65-infected RPTECs were performed at 24 h p.i. Notably, in DMSO-pretreated RPTECs, both Oki53 and Oki65 significantly decreased afadin protein levels (205 kDa) at 24 h p.i (~20% of non-infected cells), whereas nectin-2 protein level (70kDa) showed no significant decrease when compared with non-infected RPTECs (Fig 3A and 3B). Moreover, an afadin cleavage product (~170 kDa) was strongly detected in Oki53-infected RPTECs pretreated with proteasomal inhibitors, whereas this protein band was weaker in cells pretreated with Z-VAD-FMK (Fig 3A). These results suggested that Oki53 induced afadin cleavage via a caspase-dependent limited proteolysis. Furthermore, immunofluorescence analysis revealed that, under non-infected conditions, afadin was localized to both the cell membrane and nucleus, whereas nectin-2 was observed only at the cell membrane, and that infection with Oki strains caused afadin and nectin-2 displacement from the cell membrane (Fig 3C and 3D).

In Oki53-infected RPTECs, pretreatment with MG132, bortezomib, and Z-VAD-FMK significantly prevented both afadin decrease and afadin and nectin-2 displacement from the membrane. In contrast, in Oki65-infected RPTECs, these effects were only partially prevented. Specifically, the decrease in afadin protein levels by Oki65 was significantly prevented by bortezomib; however, afadin levels remained in less than half of the non-infected cells, indicating that the inhibitory effect was not sufficient to maintain the epithelial barrier integrity, which suggests that Oki53 induced afadin degradation and nectin-2 displacement via proteasomal and caspase-dependent pathways, while Oki65 disrupted the nectin–afadin complex through different mechanisms. In Oki53-infected RPTECs pretreated with proteasomal inhibitors and Z-VAD-FMK, TEER and afadin protein levels, as well as their membrane localization, were maintained at levels comparable to those in non-infected cells (Figs 2A and 3A–D). In contrast, in Oki65-infected RPTECs under the same treatment conditions, only partial preservation of these parameters was observed. These findings suggest a correlation between TEER and both the level and membrane localization of afadin, indicating that afadin may be essential for maintaining epithelial barrier integrity.

### 3.4 Differential afadin degradation and F-actin disorganization pathways lead to distinct epithelial barrier disruption mechanisms

**Fig 4:**
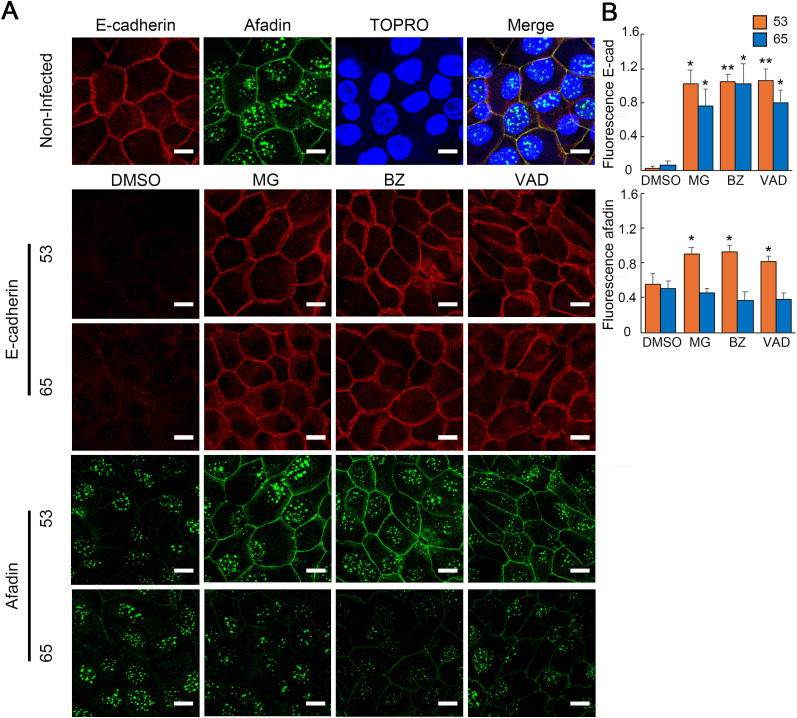
Immunofluorescence analysis using dual staining for E-cadherin (E-cad) and afadin and F-actin in RPTECs infected with Oki53 and Oki65 at 24 h post-infection. (A) Representative immunofluorescence images of afadin (green) and E-cad (red) staining in RPTECs. RPTECs were pre-treated for 30 min with DMSO or MG132 (MG), infected with clinical isolates, fixed with methanol at 24 h p.i, and processed for immunostaining. Afadin was stained with an Alexa Fluor 488-labeled antibody (green) and E-cad with a Cy3-labeled antibody (red). The cell nuclei were stained with TO-PRO-3 (blue). Scale bar: 10 μm. Merged images of E-cad, afadin, and TO-PRO-3 are provided in S5 Fig. (B) Relative fluorescence intensity of E-cad and afadin, considering the intensity of non-infected RPTECs pretreated with DMSO as 1. Each bar represents the mean ± standard deviation of three independent images. The relative fluorescence intensity of RPTEC pretreated with MG, BZ, and VAD was compared with that of RPTEC pretreated with DMSO, for each strain. *p < 0.05 and **p < 0.01.

Our results showed that the Oki strains disrupted both the E-cadherin–catenin and nectin–afadin complexes, with distinct mechanisms of afadin degradation observed between Oki53 and Oki65 strains (Figs 2 and 3). To confirm this in the same cells, immunofluorescence analysis using dual E-cadherin and afadin staining was performed in RPTECs infected with either strain at 24 h p.i. Therefore, the events observed in Figs 2D and 3C were reproduced within the same cells. That is, both Oki53 and Oki65 induced E-cadherin and afadin displacement (Fig 4). In RPTECs pretreated with MG132, bortezomib, or Z-VAD-FMK, displacement of both proteins by Oki53 was inhibited (Fig 4). In contrast, Oki65-induced displacement of E-cadherin was prevented, whereas afadin displacement remained unaffected (Fig 4). These findings suggest that Oki53 disrupts both complexes via proteasomal and caspase-dependent pathways, while Oki65 displaces the E-cadherin–catenin complex through the same pathways but affects the nectin–afadin complex via a distinct mechanism.

**Fig 5:**
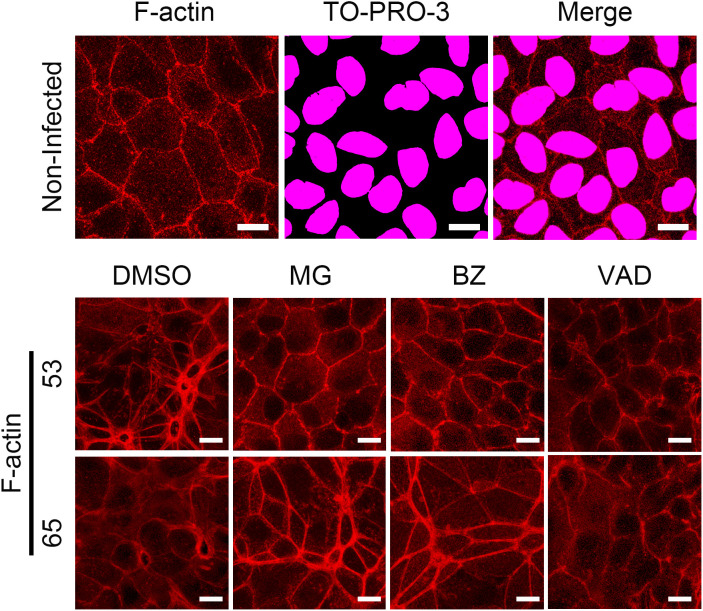
Immunofluorescence analysis for F-actin in RPTECs infected with Oki53 and Oki65 at 24 h post-infection. RPTECs were pre-treated for 30 min with DMSO, MG, bortezomib (BZ), or Z-VAD-FMK(VAD), infected with clinical isolates, fixed with 2% paraformaldehyde at 24 h p.i, and processed for immunostaining. F-actin was stained with rhodamine phalloidin (red). The cell nuclei were stained with TO-PRO-3 (magenta). Scale bar: 10 μm. Merged images of F-actin and TO-PRO-3 nuclear staining for non-infected cells with inhibitors and infected cells are provided in S6 Fig. RPTECs, renal proximal tubule epithelial cells; p.i, post-infection; DMSO, dimethyl sulfoxide.

RPTECs infected with LM reportedly cause F-actin disorganization preventable by MG132, bortezomib, and Z-VAD-FMK [13]. The nectin–afadin complex, alongside the E-cadherin–catenin complex, anchors to F-actin to form a structural network essential for maintaining intercellular junction integrity in differentiated epithelial cells, as evidenced by F-actin disorganization following afadin knockout in intestinal epithelial cells [9]. We hypothesized that afadin degradation via different pathways in Oki53 and Oki65 might also lead to F-actin disorganization and epithelial barrier disruption through distinct mechanisms. Immunofluorescence analysis of F-actin was performed in Oki53- or Oki65-infected RPTECs at 24 h p.i. Both Oki53 and Oki65 induced F-actin disorganization (Fig 5). In RPTECs pretreated with MG132, bortezomib, and Z-VAD-FMK, the F-actin disorganization caused by Oki53 was effectively prevented by proteasomal inhibitors and Z-VAD-FMK, whereas the Oki65-induced disorganization was only partially prevented by proteasomal inhibitors and Z-VAD-FMK (Fig 5). These results suggest that Oki53 hijacked proteasomal and caspase-dependent pathways, and that inhibiting these pathways preserved afadin localization at the membrane and F-actin integrity, thereby stabilizing the F-actin–AJ link and maintaining TEER. In contrast, Oki65 appeared to employ a distinct mechanism, wherein these inhibitors failed to fully preserve afadin localization and F-actin structure, potentially leading to a weakened F-actin–AJ link and insufficient TEER recovery.

## 4. Discussion

Analyzing the pathogenicity and infection mechanisms of *Leptospira* clinical isolates is challenging because it requires maintaining the isolates at low passage, as virulence is often lost through repeated subculturing [18]. In Okinawa, where human leptospirosis is endemic, close collaboration between medical institutions and the Okinawa Prefectural Institute of Health and Environment has enabled the isolation and preservation of low-passage clinical strains, facilitating this research. In a previous study, which analyzed long-term stocked clinical strains, one of the two isolates from Okinawa showed no significant TEER reduction [19]. Thus, it remains unclear whether this was due to the strain becoming avirulent during several *in vitro* passages and/or long-term storage in the laboratory, or the inability of some clinical strains to induce TEER decline in human RPTECs. In this study, 11 clinical isolates from 2023 were stored at -80°C, and at low passages immediately after optimal growth. This study is the first to use passage-controlled clinical isolates in Okinawa, Japan, for investigation of infection mechanisms. Notably, all 11 clinical strains of *Leptospira interrogans* isolated from patients in 2023 induced TEER reduction (Fig 1). These findings indicate that epithelial barrier disruption is a common effect of *L. interrogans,* supporting the use of TEER as a reliable indicator of its pathogenic activity. Barocchi *et al*. reported that *L. interrogans* did not affect TEER in MDCK cells at 4 h p.i. [20]. In our experimental design using RPTECs, the TEER decrease was observed at 6 h p.i, which suggested that the virulence factors responsible for epithelial barrier disruption might be expressed after leptospiral interaction with the eukaryotic cells [21]. Conversely, an increase in TEER was observed in *L. biflexa*-infected RPTECs (Fig 1). *Bifidobacterium longum* induces a dose-dependent increase in TEER via toll-like receptor signaling [22]. Nonpathogenic *Leptospira* may enhance TEER by inducing protective responses in RPTECs; however, further studies are required to elucidate the underlying mechanism. As a limitation, clinical data such as biochemical markers of kidney injury were not provided by the medical institutions for the 11 isolates used in this study, and therefore correlations with TEER values could not be assessed.

This study is the first to demonstrate that *L. interrogans* induces afadin protein level reduction and disrupts its localization at the plasma membrane (Fig 3A–D). Similar phenomena have been reported for other bacterial pathogens. For example, *Aeromonas sobria* produces a serine protease (ASP) that selectively degrades afadin and nectin-2 in human intestinal epithelial cells, resulting in reduced TEER and compromised epithelial barrier function [23]. *Helicobacter pylori* infection also induces afadin degradation, decreases TEER, increases paracellular permeability, disrupts the actin cytoskeleton, and ultimately promotes epithelial-to-mesenchymal transition [24]. Furthermore, RNAi-mediated afadin silencing leads to the displacement of junctional proteins, including E-cadherin and β-catenin from cell–cell contacts, increased paracellular permeability, and actin cytoskeleton disorganization, all contributing to impaired junctional integrity and enhanced cell motility and invasion [24]. In this study, the TEER reduction level induced by Oki53 and Oki65 strains that was prevented by pretreatment with proteasomal and caspase inhibitors in RPTECs (Fig 2A) strongly correlated with the degree of afadin protein degradation (Fig 3A and 3B). These findings suggested that afadin degradation plays a significant role in leptospiral infection strategies, particularly by contributing to epithelial barrier disruption. Notably, afadin is the sole adaptor protein that binds directly to nectin. In an afadin-knockout mouse, nectin-2 and -3 were displaced from the cell membrane, whereas afadin localization was preserved in a nectin-2/3-knockout mouse, suggesting that afadin localization at adherens junctions was nectin-independent, whereas nectin localization depends on afadin [10]. In this study, Oki53 and Oki65 infection induced nectin-2 displacement from the membrane and afadin degradation in RPTECs (Fig 3A–D). Notably, these effects were inhibited by pretreatment with proteasomal inhibitors and Z-VAD-FMK in Oki53-infected cells, but not in Oki65-infected cells (Fig 3A–D). These findings suggest that afadin degradation might be responsible for nectin-2 displacement from the plasma membrane. In contrast, *L. interrogans* serovar Copenhageni did not affect nectin localization in HMEC-1 endothelial cells at 24 h p.i. [25]. This discrepancy might originate from differences in cell type, indicating that *Leptospira* does not disrupt afadin in endothelial cells. In previous studies, *Leptospira* infection has been shown to induce endocytosis of E-cadherin [12]. In the present study, E-cadherin, α-catenin, and Nectin-2 were displaced from the cell membrane upon infection, but their protein levels were not decreased in whole-cell lysates (Figs 2 and 3). These findings suggest that the junctional proteins remained within the cells rather than being degraded. Further studies will be required to determine their exact intracellular localization.

Bacterial strains of the same species can vary significantly in pathogenic potential due to differences in virulence factors, regulatory networks, or host interactions. For instance, distinct strains of *Helicobacter pylori* differ in the presence or absence of cytotoxin-associated gene A (CagA) activity, resulting in differential epithelial responses and disease severity [26]. Similarly, in *Escherichia coli*, specific pathotypes such as enterohemorrhagic *Escherichia coli* and enteropathogenic *Escherichia coli* markedly differ in the mechanisms by which they disrupt host epithelial barriers, largely due to the presence or absence of distinct virulence genes [27]. Notably, *L. interrogans* serovar Manilae strain UP-MMC-NIID caused severe distortion of tubular cell arrangement and renal tubules disruption in kidney tissues, as well as hemorrhage in lung tissues, ultimately leading to lethality in hamsters, while *L. interrogans* serovar Hebdomadis strain OP84 failed to cause death [14,28]. As leptospiral differential virulence factors, a family of 12 secreted virulence-modifying proteins (VMPs) in *L. interrogans* reportedly induces F-actin disorganization and increases paracellular permeability to facilitate tissue invasion [29,30]. In this study, both Oki53 and Oki65 induced AJ disruption and F-actin disorganization in RPTECs, with TEER disruption levels closely mirroring the degree of F-actin disorganization under various inhibitor treatments (Figs 2A and 5). F-actin disorganization promotes clathrin-dependent endocytosis of E-cadherin [31] and causes α-catenin to dissociate from the membrane [32]. These findings suggest that pathogenic *Leptospira* degrade key AJ components, such as afadin, and induce F-actin disorganization via secreted bacterial factors such as VMPs and/or hijack eukaryotic proteolytic systems to effectively disrupt the epithelial barrier. Notably, the sequences and expression levels of VMPs vary among *Leptospira* lineages [33]. Moreover, Oki53 induced epithelial barrier disruption via a proteasome- and caspase-dependent pathway, whereas Oki65 appeared to involve an additional mechanism, as the disruption was only partially inhibited (Fig 2A). These findings suggest that these strain-dependent differences in AJ disruption mechanisms may be attributed to variations in VMP type and expression. Although the direct mechanistic relationships remain unclear, VMP-induced disruption of F-actin architecture, activation of proteasomal and/or caspase pathways, and the degradation of junctional components such as afadin are possibly interconnected events. These strain-dependent variations could potentially influence disease kinetics and clinical outcomes, highlighting the need for further studies to identify the molecular determinants underlying these differences, which may also serve as potential therapeutic targets. A limitation of this study is that, among the 11 clinical isolates tested, not all strains showed a significant reduction in TEER at 24 hours post-infection, the earliest time point before inhibitor-induced cytotoxicity becomes evident. Expanding the analysis to a broader panel of isolates representing different lineages and applying additional experimental approaches would help clarify whether the distinct mechanisms disrupting the nectin–afadin–F-actin and E-cadherin–catenin–F-actin complexes are strain-specific, serogroup-specific, or associated with particular genetic lineages.

Jarisch–Herxheimer reactions (JHR), which result from the abrupt release of endotoxins following antibiotic-induced bacterial lysis, are a well-known complication during leptospirosis treatment [34]. In Okinawa, JHR has been reported in >80% of patients with leptospirosis, with occasional fatal outcomes [35,36]. In addition, our study is the first to demonstrate that a proteasomal inhibitor can fully prevent epithelial barrier disruption caused by a clinical *Leptospira* strain (Oki53). Notably, bortezomib, a proteasomal inhibitor already approved for clinical use, may serve as a potential alternative to conventional antibiotics [37–39], offering the added benefit of possibly avoiding JHR. Although its efficacy varies among strains, further animal studies are warranted to assess the therapeutic potential of bortezomib against leptospiral infection *in vivo*. Although Z-VAD-FMK effectively prevented epithelial barrier disruption caused by Oki53, it is a pan-caspase inhibitor and does not identify the specific caspases involved. For instance, in *Helicobacter pylori* infection, caspase-3 activation causes TEER reduction and E-cadherin disorganization, which can be prevented by the caspase-3–specific inhibitor Z-DEVD-FMK [40]. However, Z-DEVD-FMK does not inhibit TEER reduction or E-cadherin disorganization in *Leptospira interrogans* serovar Manilae infection [13]. Future studies should use specific inhibitors to determine the caspases exploited by *Leptospira*.

**Fig 6:**
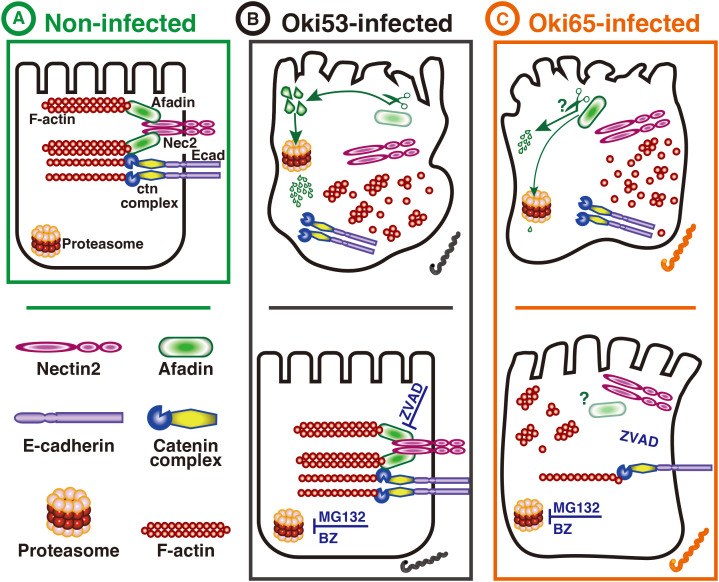
Hypothetical model representing the distinct strategies of epithelial barrier disruption by Oki strains. (A) Non-infected RPTECs. E-cad and Nectin-2 are located at the plasma membrane and linked to the F-actin cytoskeleton through a-ctn and afadin, respectively. (B) Oki53-infected RPTECs (24 h p.i.). (C) Oki65-infected RPTECs (24 h p.i.). Upper panels (DMSO-pretreated RPTECs). The F-actin cytoskeleton is disorganized, the E-cad/ctn complex and Nectin-2 are displaced from the plasma membrane, and afadin is degraded. Lower panels (inhibitors-pretreated RPTECs): (B) In Oki53-infected RPTECs: afadin degradation is prevented, the E-cad/ctn and Nectin/afadin complexes are not displaced from the plasma membrane, and the F-actin cytoskeleton structure is not disturbed; (C) In Oki65-infected RPTECs: E-cad/ctn complex is not displaced from the membrane; however, afadin degradation and Nectin-2 displacement from the membrane are not prevented, thus, the F-actin cytoskeleton structure is partially disturbed. RPTECs, renal proximal tubule epithelial cells; p.i, post-infection; DMSO, dimethyl sulfoxide.

In conclusion, this study demonstrated that clinical isolates of *L. interrogans* disrupt the renal epithelial barrier through diverse, strain-dependent mechanisms with differing effects on the nectin-afadin-F-actin link and the E-cadherin-catenin-F-actin link (Fig 6). These findings highlight the importance of analyzing multiple clinical strains to understand the variability in pathogenic strategies, which may be influenced by differences in virulence factors, host interactions, or genetic backgrounds.

## Supporting information

S1 Fig Uncropped blots corresponding to Fig 2B, provided for assessment of band specificity and loading consistency. (TIF)

S2 Fig Representative merged immunofluorescence images of E-cadherin, α-catenin, and TO-PRO-3. (TIF)

S3 Fig Uncropped blots corresponding to Fig 3A, provided for assessment of band specificity and loading consistency. (TIF)

S4 Fig Representative merged immunofluorescence images of afadin with TO-PRO-3, and nectin-2 with TO-PRO-3. (TIF)

S5 Fig Representative merged immunofluorescence images of E-cadherin, afadin, and TO-PRO-3. (TIF)

S6 Fig Representative merged immunofluorescence images of F-actin and TO-PRO-3. (TIF)
