# Supplementary figures and images for: Distinct strategies of epithelial cell barrier disruption by Leptospira interrogans isolated from human patients in Okinawa, Japan
Source: PLoS Negl Trop Dis. 2025 Nov 4;19(11):e0013693. doi: 10.1371/journal.pntd.0013693 (PMC12604759; doi:10.1371/journal.pntd.0013693)

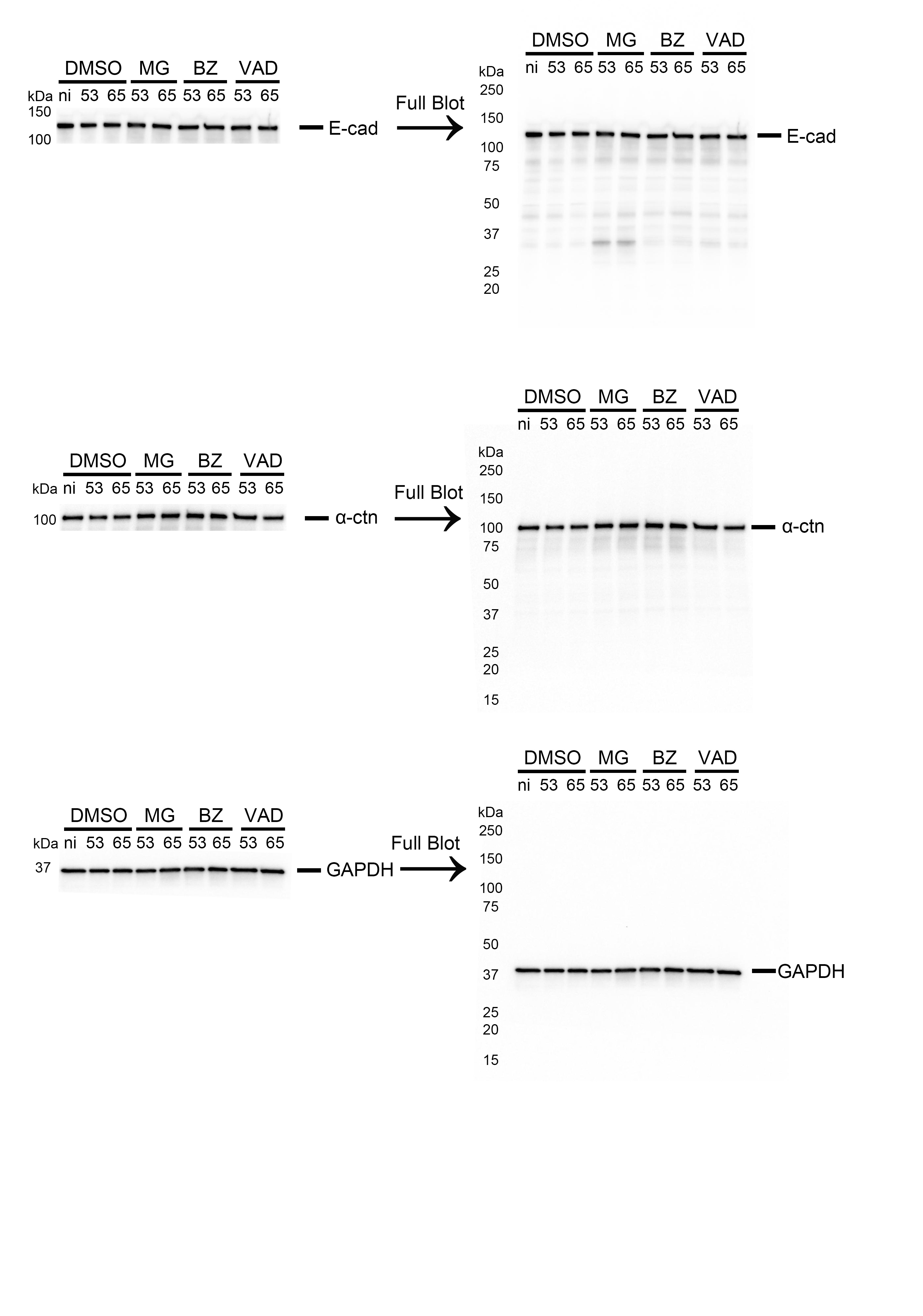

Supplement: S1 Fig — (TIF) [file pntd.0013693.s001.tif]

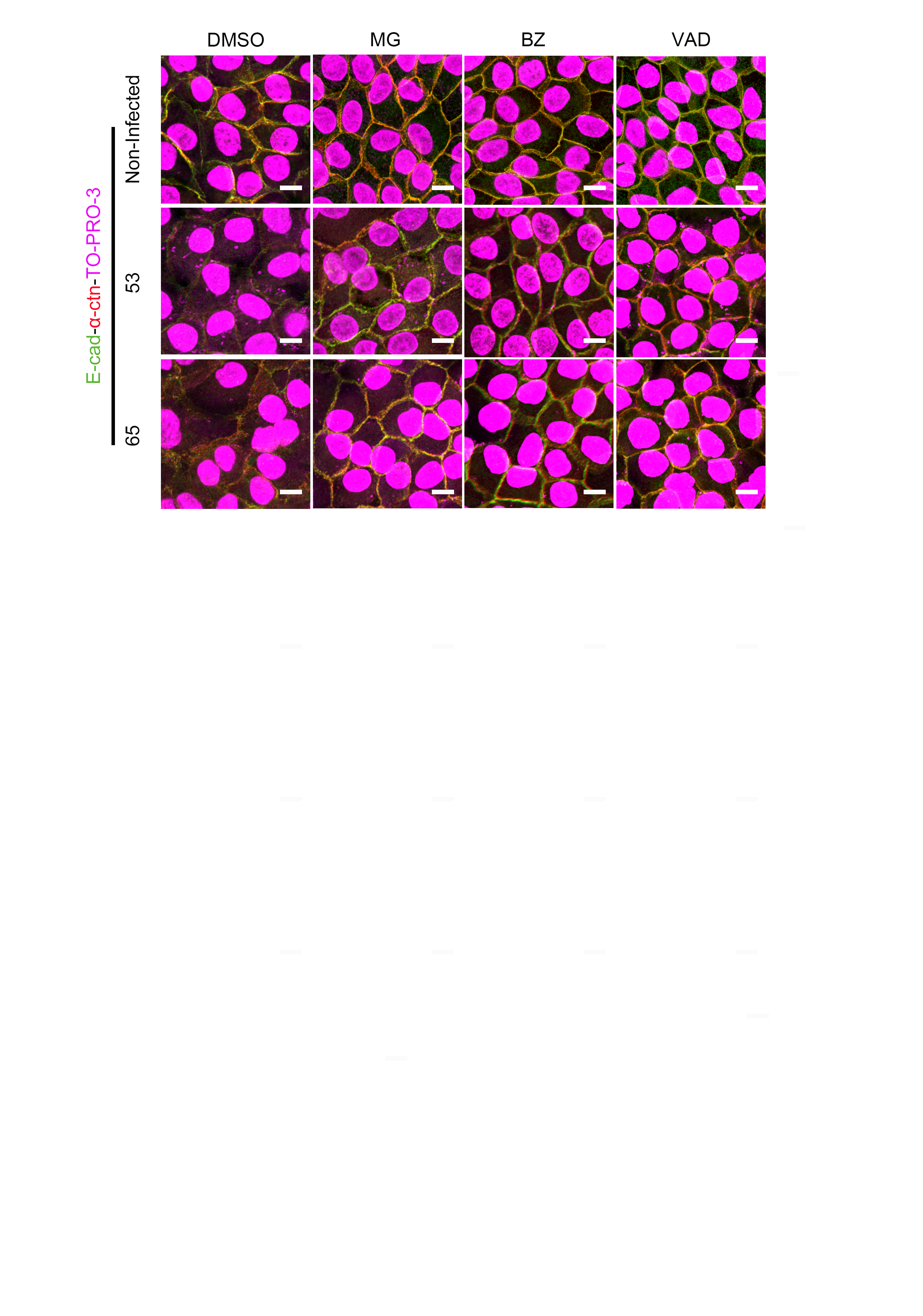

Supplement: S2 Fig — (TIF) [file pntd.0013693.s002.tif]

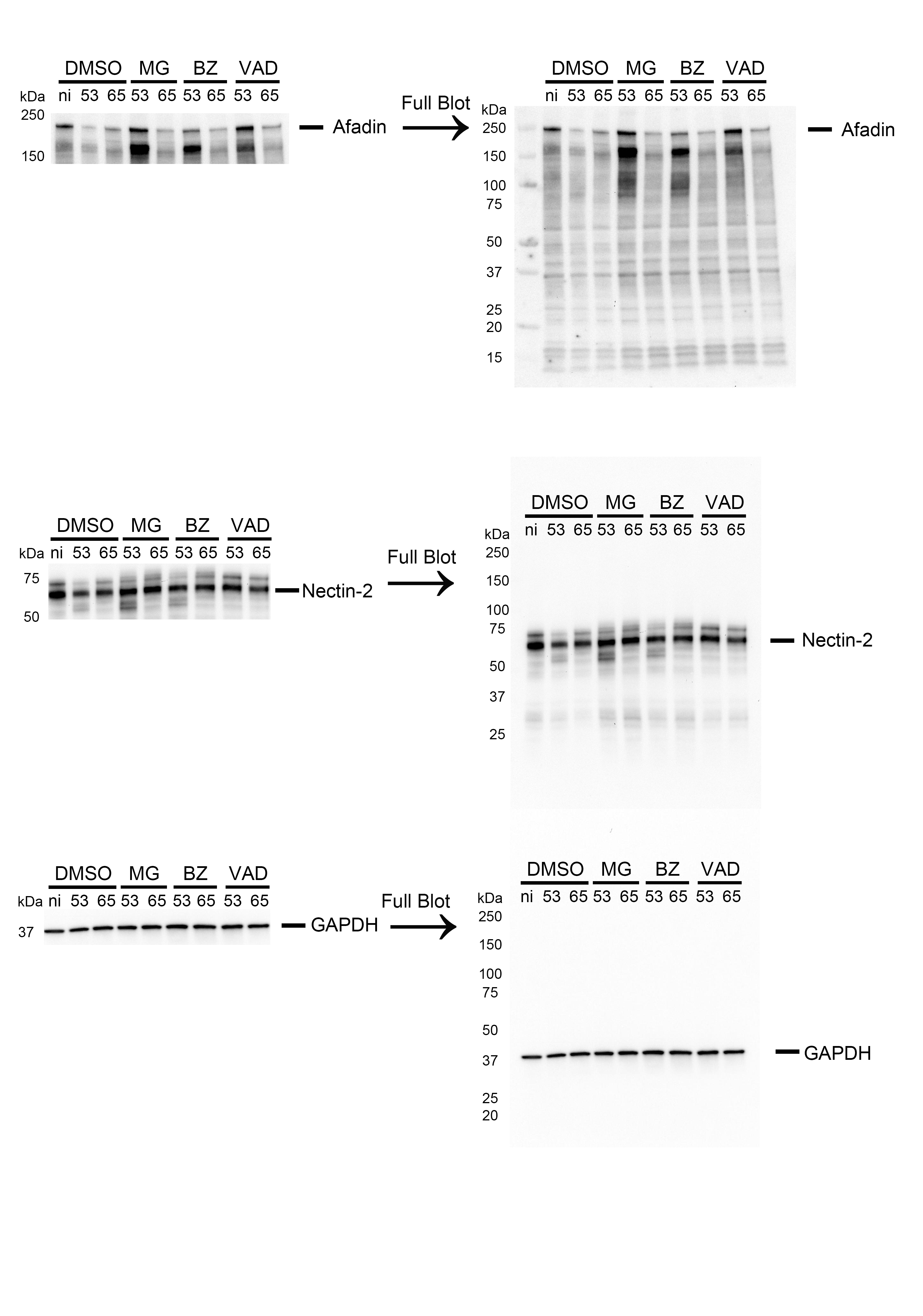

Supplement: S3 Fig — (TIF) [file pntd.0013693.s003.tif]

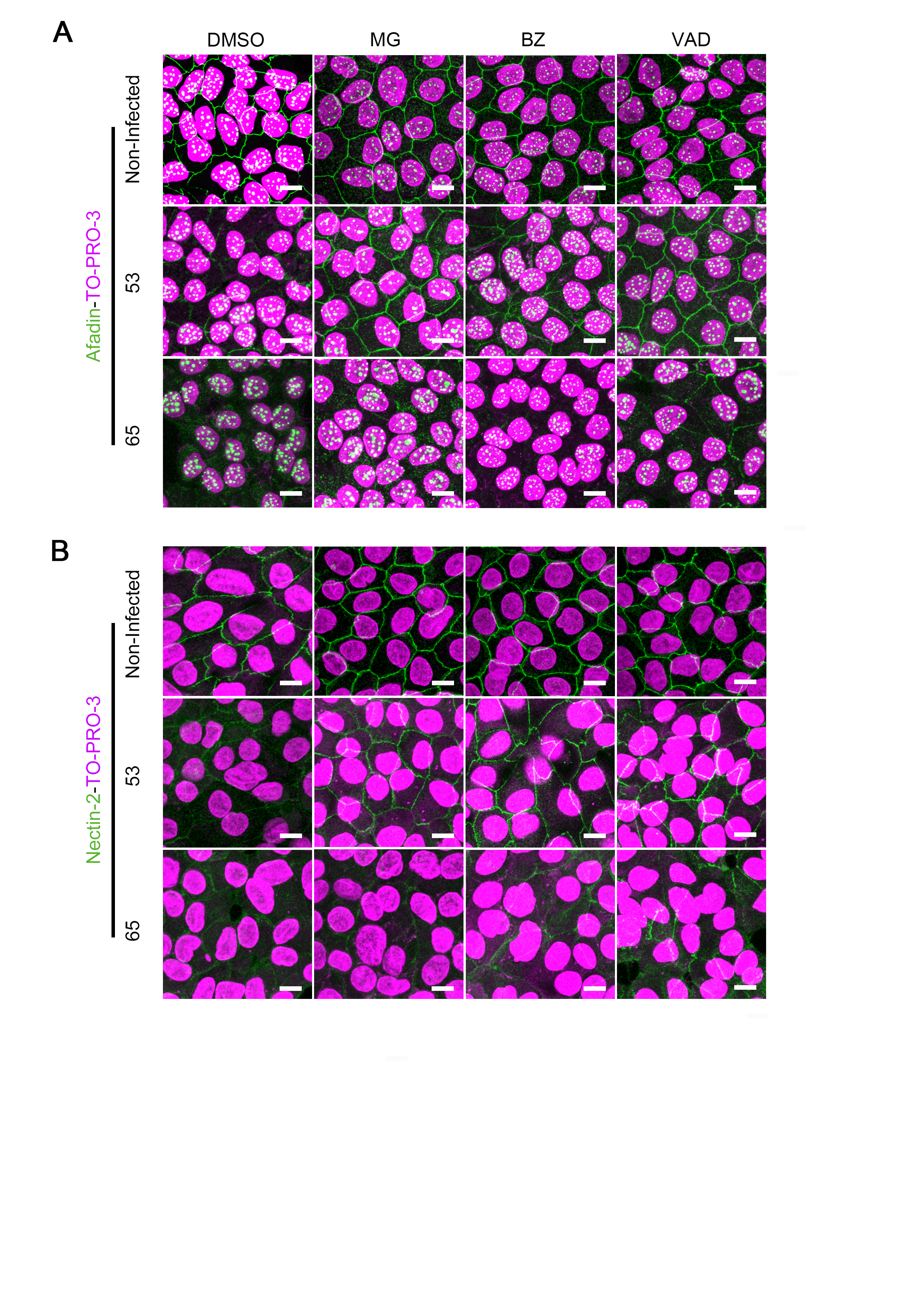

Supplement: S4 Fig — (TIF) [file pntd.0013693.s004.tif]

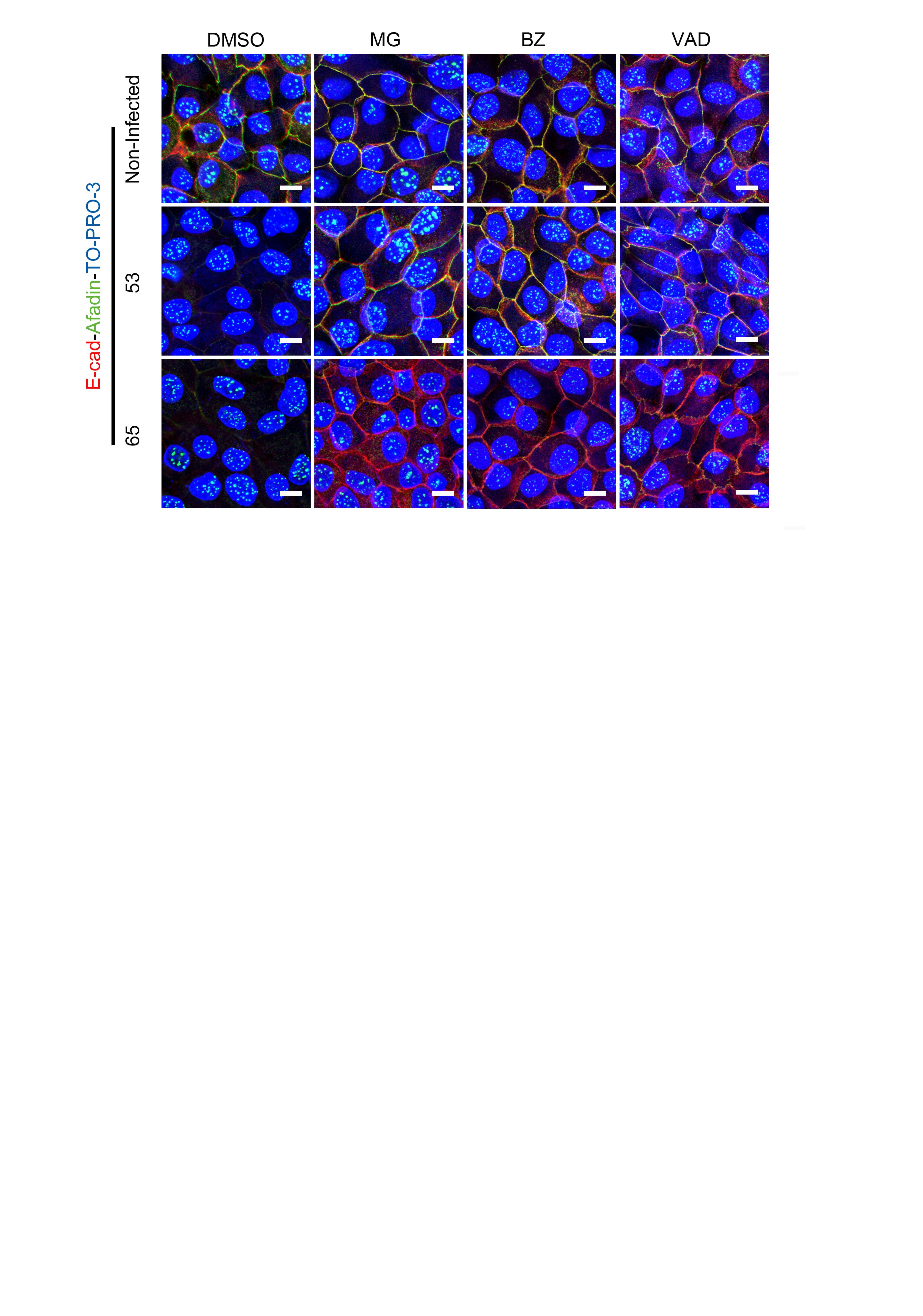

Supplement: S5 Fig — (TIF) [file pntd.0013693.s005.tif]

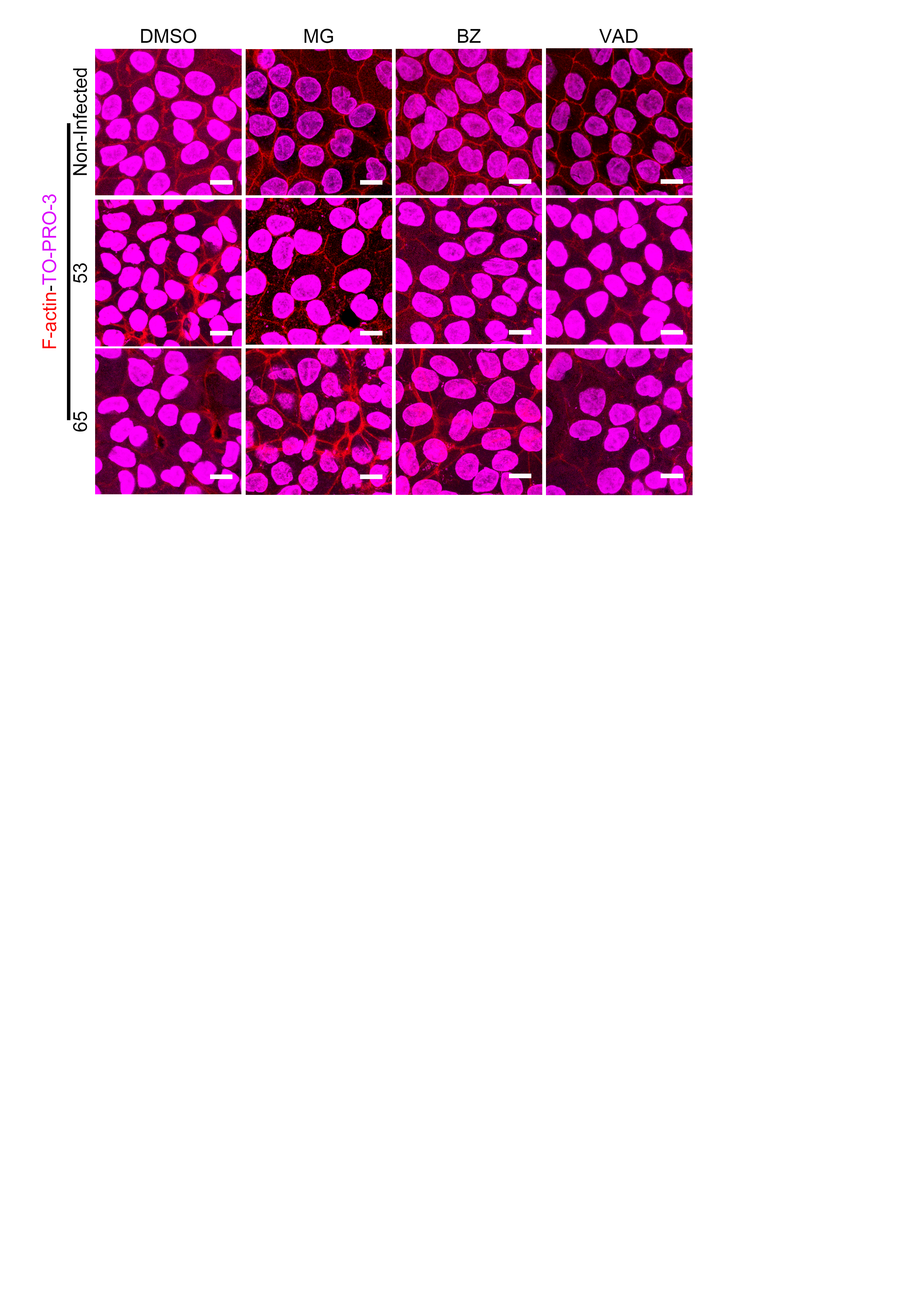

Supplement: S6 Fig — (TIF) [file pntd.0013693.s006.tif]
